# Water Quality Index for measuring drinking water quality in rural Bangladesh: a cross-sectional study

**DOI:** 10.1186/s41043-016-0041-5

**Published:** 2016-02-09

**Authors:** Tahera Akter, Fatema Tuz Jhohura, Fahmida Akter, Tridib Roy Chowdhury, Sabuj Kanti Mistry, Digbijoy Dey, Milan Kanti Barua, Md Akramul Islam, Mahfuzar Rahman

**Affiliations:** 1 BRAC Research and Evaluation Division, BRAC Centre, 75 Mohakhali, Dhaka, 1212 Bangladesh; 2 BRAC Tuberculosis Programme, BRAC Centre, 75 Mohakhali, Dhaka, 1212 Bangladesh; 3 BRAC Water, Sanitation and Hygiene Programme, BRAC Centre, 75 Mohakhali, Dhaka, 1212 Bangladesh

**Keywords:** Water quality index, Chemical parameters, WASH program, BRAC, Bangladesh

## Abstract

**Background:**

Public health is at risk due to chemical contaminants in drinking water which may have immediate health consequences. Drinking water sources are susceptible to pollutants depending on geological conditions and agricultural, industrial, and other man-made activities. Ensuring the safety of drinking water is, therefore, a growing problem. To assess drinking water quality, we measured multiple chemical parameters in drinking water samples from across Bangladesh with the aim of improving public health interventions.

**Methods:**

In this cross-sectional study conducted in 24 randomly selected *upazilas*, arsenic was measured in drinking water in the field using an arsenic testing kit and a sub-sample was validated in the laboratory. Water samples were collected to test water pH in the laboratory as well as a sub-sample of collected drinking water was tested for water pH using a portable pH meter. For laboratory testing of other chemical parameters, iron, manganese, and salinity, drinking water samples were collected from 12 out of 24 *upazilas*.

**Results:**

Drinking water at sample sites was slightly alkaline (pH 7.4 ± 0.4) but within acceptable limits. Manganese concentrations varied from 0.1 to 5.5 mg/L with a median value of 0.2 mg/L. The median iron concentrations in water exceeded WHO standards (0.3 mg/L) at most of the sample sites and exceeded Bangladesh standards (1.0 mg/L) at a few sample sites. Salinity was relatively higher in coastal districts. After laboratory confirmation, arsenic concentrations were found higher in Shibchar (Madaripur) and Alfadanga (Faridpur) compared to other sample sites exceeding WHO standard (0.01 mg/L). Of the total sampling sites, 33 % had good-quality water for drinking based on the Water Quality Index (WQI). However, the majority of the households (67 %) used poor-quality drinking water.

**Conclusions:**

Higher values of iron, manganese, and arsenic reduced drinking water quality. Awareness raising on chemical contents in drinking water at household level is required to improve public health.

## Background

Quality of drinking water indicates water acceptability for human consumption. Water quality depends on water composition influenced by natural process and human activities. Water quality is characterized on the basis of water parameters (physical, chemical, and microbiological), and human health is at risk if values exceed acceptable limits [1–3]. Various agencies such as the World Health Organization (WHO) and Centers for Disease Control (CDC) set exposure standards or safe limits of chemical contaminants in drinking water. A common perception about water is that clean water is good-quality water indicating knowledge gap about the presence of these substances in water. Ensuring availability and sustainable management of good-quality water is set as one of the Sustainable Development Goals (SDGs) and is a challenge for policy makers and Water, Sanitation and Hygiene (WASH) practitioners, particularly in the face of changing climatic conditions, increasing populations, poverty, and the negative effects of human development.

Water Quality Index (WQI) is considered as the most effective method of measuring water quality. A number of water quality parameters are included in a mathematical equation to rate water quality, determining the suitability of water for drinking [4]. The index was first developed by Horton in 1965 to measure water quality by using 10 most regularly used water parameters. The method was subsequently modified by different experts. These indices used water quality parameters which vary by number and types. The weights in each parameter are based on its respective standards, and the assigned weight indicates the parameter’s significance and impacts on the index. A usual WQI method follows three steps which include (1) selection of parameters, (2) determination of quality function for each parameter, and (3) aggregation through mathematical equation [5]. The index provides a single number that represents overall water quality at a certain location and time based on some water parameters. The index enables comparison between different sampling sites. WQI simplifies a complex dataset into easily understandable and usable information. The water quality classification system used in the WQI denotes how suitable water is for drinking. The single-value output of this index, derived from several parameters, provides important information about water quality that is easily interpretable, even by lay people [6]. In a resource-poor country like Bangladesh where ensuring availability and sustainable management of water is one of the challenging areas towards development. The present study embraced weighted arithmetic WQI method to deliver water quality information to WASH practitioners. One of the merits of this method is that a less number of parameters are required to compare water quality for certain use [5].

The WASH program of the Bangladesh Rural Advancement Committee (BRAC) has provided interventions in 250 *upazilas* in Bangladesh since 2006 with the aim of improving the health of the rural poor. The BRAC WASH program selects intervention areas on the basis of some criteria such as high poverty rate, poor sanitation coverage, and lack of access to safe water due to high arsenic, salinity, and other contaminants [7]. The program has adopted a holistic approach integrating water, sanitation, and hygiene components. The water component promotes use of safe water through a number of activities: (1) deep tubewell installation in arsenic-affected areas; (2) loan to construct tubewell platform in order to protect groundwater from pollutions; and (3) water quality testing [8]. Besides, awareness building and behavioral change remain at the core of the WASH program [9] to improve health and hygiene of the rural poor. The types of interventions vary according to households’ economic status.

Earlier, we conducted a number of studies on water- and hygiene-related issues in intervention areas, such as use of tubewell water and water safety practices [8], women in water hygiene [10], and knowledge gap on hygiene and safe water [7]. Some impeding factors towards access to safe drinking are poverty, unhygienic sanitation practices, low groundwater levels, and impacts of natural hazards (e.g., arsenic, salinity, extreme weather events) [11]. The program assessed water safety in a crude way by some proxy indicators such as awareness on brick-built tubewell platform, its cleanliness, and no waterlogging at the bottom of the tubewell. To our knowledge, the present study on water quality assessment based on some water parameters has been the first study conducted for the BRAC WASH program. We aimed through this research to understand households’ exposure to these water parameters according to their background characteristics which might have programmatic implications in the future. The present study measures drinking water quality with the application of weighted arithmetic WQI method based on some chemical parameters. These parameters used for drinking water quality assessment were selected as the requirement of BRAC WASH program. The relevance of the present study lies in programmatic implications by providing evidence-based and useful information on drinking water quality in a simple way. We expect that the findings will help in designing program interventions to ensure safe drinking water either by raising awareness about chemical contamination of water or by improving water quality through provision of hardware supply.

## Methods

### Study design and area

**Fig. 1:**
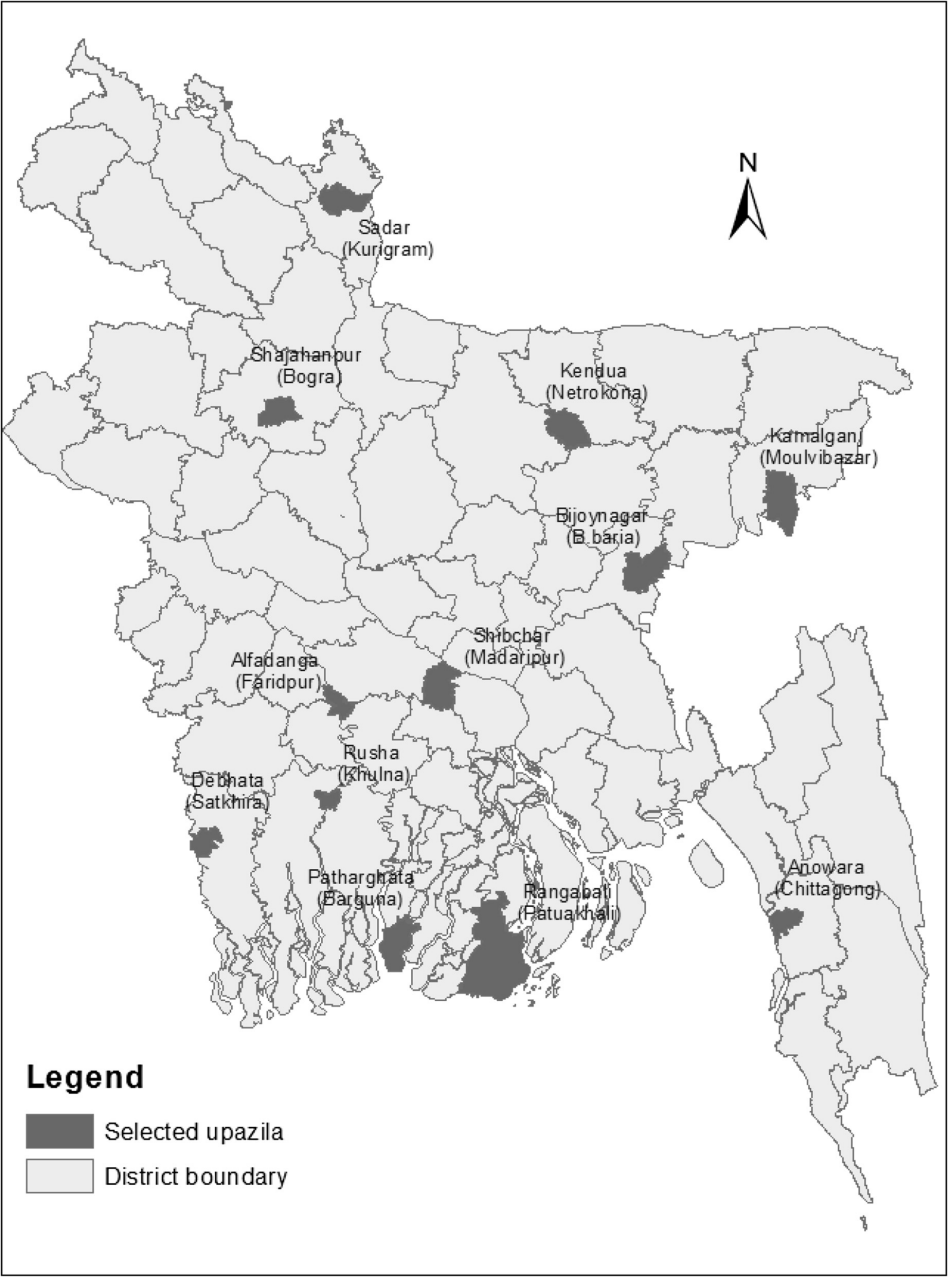
Selected upazila for water sample collection

This study was part of our research on “The status of household WASH behaviors in rural Bangladesh,” conducted in 24 randomly selected *upazilas* (5 % of total). The current study on the assessment of drinking water quality used a cross-sectional study design and was conducted in 12 out of 24 *upazilas* across the country: Alfadanga (Faridpur), Kendua (Netrokona), Shibchar (Madaripur), Rupsha (Khulna), Debhata (Satkhira), Patharghata (Barguna), Rangabali (Patuakhali), Anwara (Chittagong), Bijoynagar (Brahmanbaria), Shajahanpur (Bogra), Kamalganj (Moulvibazar), and Kurigram Sadar (Kurigram) (Fig. 1).

### Study procedure

A total of 960 households from 24 *upazilas* (40 households in each *upazila*) were randomly selected for socioeconomic survey and arsenic test on the spot using test kit at household level. Twelve out of 24 *upazilas* were considered to collect water samples from drinking water sources and to test chemical parameters in the laboratory. A total of 542 water samples were collected from 293 randomly selected households. In each *upazila*, 20 out of 40 households were initially selected for water samples collection. However, the total number of samples varied due to some reasons: (1) samples collected from shared tubewells increased the number of households; and (2) a few water samples were discarded due to label numbers being washed away, rendering them unidentifiable. Of the total water samples collected, same samples (293 samples) were used to test both iron and manganese levels in water. Similarly, the remaining 249 water samples were used to test both pH and salinity (Table 1).

**Table 1:** Sample distribution

	Spot test	Laboratory test				
Chemical parameter	Arsenic (As)	Arsenic (As)	Manganese (Mn)	Iron (Fe)	pH	Salinity (NaCl)
Households visited	960	293				
Sample tested	645	64	293	293	249	249
Total sample	645	542				

A total of 36 research assistants were recruited and grouped into 12 to collect water samples from selected *upazilas*. They were trained intensively for 3 days and a field test was conducted nearby Dhaka prior to actual field survey. Groundwater samples from each tubewell were collected after 2 min of pumping in order to obtain deep water as the test sample. The water samples were collected in 100-mL pre-washed bottles with watertight seals. The collected water samples were labeled with the household identification number and name of water parameters.

### Arsenic test on the spot

A total of 960 households from 24 *upazilas* were visited for arsenic testing in the field. Simultaneously, a pre-tested structured questionnaire was used to obtain household-level information on socioeconomic condition. Of the total households visited, 66 and 31 % households used shallow (<300 ft) and deep (≥300 ft) tubewells for collecting drinking water, respectively. Out of the total households using tubewells, 645 tubewells (424 shallow and 221 deep tubewells) were tested on the spot for arsenic using the “econo quick (EQ) arsenic test kit.” The nature of EQ kit reading is quantitative. A color chart in a scale of values between 0.0 and 1.0 mg/L was used to record the arsenic status of water samples tested in the field. The EQ kit was preferred to use in field test because of its high accuracy (about 90 %) of measuring arsenic status of the tubewells [12]. Drinking water sources of the remaining households (33 %) were not considered for arsenic testing for various reasons: tubewells of 29 % of households had already been tested and declared arsenic free (0.0 mg/L) in the recent past, and 4 % used pond water for drinking and were excluded from arsenic testing.

### Testing of water samples using pH meter

Acid-base balance is assessed by the pH value of water [13]. A controlled water pH is suggested in WHO guidelines to reduce adverse health consequences. According to the WHO guidelines of drinking water quality, exposure to both high and low pH values causes irritation the eyes, skin, and mucous membrane for humans [14]. Here, 123 water samples were randomly selected from the total samples collected to test the pH. A portable pH meter (model PHS-25) was used in the BRAC head office to test the pH. pH meter operating instructions were carefully followed: the meter was first calibrated by putting the electrode into standard buffer solutions of pH 6.86 and pH 4.00 at set temperature prior to being washed with distilled water and sample measurement.

### Methods used at laboratory for measuring parameters

Arsenic results measured in the field using the testing kits were verified in the laboratory. About 10 % of collected water samples were picked at random for laboratory validation. pH meter values were similarly crosschecked in the laboratory for validation. The other chemical contents (e.g., iron, manganese, and salinity) in water samples were also measured in the Water Quality Testing Laboratory of the NGO Forum for Public Health. The water samples were analyzed by flow-injection hydride generation atomic absorption spectrometry (FI-HG-AAS) method for arsenic detection. The minimum detection level for this method was 3 (μg/L). Total arsenic was measured. The efficiency of field kits used by NGO Forum for arsenic testing was reported to have low failure rate (11 % for Merck kit, 6.2 % for NIPSOM), supporting high kit’s performance in arsenic testing [15]. Manganese was analyzed in Flame (air-acetylene) AAS. The minimum detection limit of this method was 0.01 ppm. For both arsenic and manganese, AAS of Shimadzu (model: AA-6300) was used at the laboratory. Iron was analyzed by phenanthroline method using UV-visible spectrophotometer where iron was brought into a solution, reduce to a ferrous state by treating with acid and hydroxylamine and 1, 10-phenanthroline. The minimum detection limit of this method was 0.05 ppm. UV-visible spectrophotometer of Shimadzu (model: UV-1601) was used at the laboratory. Salinity was measured at the laboratory by conductivity method using an appropriate electrode.

### Data analysis

Descriptive statistics were used to analyze the mean, median, standard deviation, interquartile range (IQR), and frequency distribution of each parameter. The households’ wealth index was developed based on ownership of valued items. Bangladesh and WHO guideline standards were considered in the evaluation of the number of household members exceeding acceptable drinking water limits. The exposure level of household members was analyzed by their background characteristics which included age, sex, education, economic status, and media access at home, NGO membership, wealth index, and type of water sources used. The households were classified as ultra-poor, poor, and non-poor as per the following criteria of the BRAC WASH program: households that owned less than 404.7 m^2^ of land, had no fixed source of income, or were headed by a female were classified as “ultra-poor”; households with land holdings between 404.7 and 4047 m^2^ and/or sold manual labor for a living were classified as “poor”; and households that did not fall into either of the above categories were classified as “non-poor.” Wealth index was developed based on the ownership of valued items at household level.

### Weighted arithmetic Water Quality Index (WQI) method

The weighted arithmetic WQI method [16, 17] was applied to assess water suitability for drinking purposes. In this method, water quality rating scale, relative weight, and overall WQI were calculated by the following formulae:

$$ {\boldsymbol{q}}_{\boldsymbol{i}} = \left({\boldsymbol{C}}_{\boldsymbol{i}}/{\boldsymbol{S}}_{\boldsymbol{i}}\right) \times 100 $$

where *q*_*i*_, *C*_*i*_, and *S*_*i*_ indicated quality rating scale, concentration of *i* parameter, and standard value of *i* parameter, respectively.

Relative weight was calculated by

$$ {\boldsymbol{w}}_{\boldsymbol{i}}=1/{\boldsymbol{S}}_{\boldsymbol{i},} $$

where the standard value of the *i* parameter is inversely proportional to the relative weight.

Finally, overall WQI was calculated according to the following expression:

$$ \mathrm{W}\mathrm{Q}\mathrm{I} = {\displaystyle \sum {\boldsymbol{q}}_{\boldsymbol{i}}{\boldsymbol{w}}_{\boldsymbol{i}}}/{\displaystyle \sum {\boldsymbol{w}}_{\boldsymbol{i}}} $$

### Ethics statement

The research protocol was approved by the ethical review committee of James P Grant School of Public Health, BRAC University.

## Results

### Demographic and socioeconomic profile of households

The background characteristics of households from whom water samples were collected for laboratory testing are shown in Table 2. A total of 293 households comprising 1491 members were included in the analysis. The proportions of male and female household members were 51 and 49 %, respectively. Over half of members had higher secondary education and above followed by secondary (22 %), primary (20 %), and no schooling (8 %). Members belonged to poor (37 %), ultra-poor (30 %), and non-poor (32 %) economic groups. The study participants represented six divisions (highest administrative boundary of Bangladesh) including Dhaka and Khulna (8 % in each), Chittagong and Barisal (about 28 % in each), Rajshahi (11 %), and Sylhet (18 %). The majority had access to media (radio and/or television) at home (51 %), and 55 % of the members had no NGO membership.

**Table 2:** Demographic and socioeconomic characteristics of households

Characteristics	Frequency (HH^a^ members)	Percentage (%)
Sex		
Male	757	50.8
Female	734	49.2
Age(years)		
≤4	106	7.2
5–20	516	34.9
21–40	455	30.8
41–60	300	20.3
≥61	102	6.9
Educational level		
No schooling	62	7.6
Primary	166	20.3
Secondary	177	21.6
Higher secondary and above	414	50.5
Division		
Dhaka	115	7.7
Chittagong	414	27.8
Rajshahi	160	10.7
Khulna	121	8.1
Barisal	411	27.6
Sylhet	270	18.1
Occupation		
Agriculture	123	9.0
Laborer (skilled/unskilled)	123	9.0
Housewife/homestead task	408	29.9
Service/professional	87	6.4
Business	88	6.5
Student	427	31.3
Unemployed/disabled	70	5.1
Others	38	2.8
Household economic status		
Ultra-poor	452	30.3
Poor	557	37.4
Non-poor	482	32.3
Marital status		
Unmarried	692	46.4
Married	728	48.8
Widow/separated/divorced	71	4.8
Access to media at home		
No access to media	730	49.0
Access to media	761	51.0
NGO membership		
No membership	816	54.7
Member of any NGO	675	45.3
Wealth index		
Lowest	342	23.1
Second	301	20.3
Middle	265	17.9
Fourth	262	17.7
Highest	313	21.1
Total	1491	100

^a^Household

### pH levels in the drinking water

The median of pH value was 7.4, while IQR values at different sample sites varied between 0.2 and 0.4, respectively. The highest frequency value was pH 7.4 (34 %) followed by pH 7.2 (13 %) and pH 7.6 (9 %). pH values at selected sites ranged between 6.6 and 8.4 (Table 3), within acceptable limits (6.5–8.5). The mean pH values in both shallow (7.5 ± 0.4) and deep tubewells (7.4 ± 0.3) varied, but median value was found the same in both types (7.4 mg/L).

**Table 3:** Regional variation in the values of chemical parameters of drinking water

		Chemical parameter				
Sample site		pH	Manganese (Mn) (mg/L)	Iron (Fe) (mg/L)	Salinity (NaCl) (mg/L)	Arsenic (As) (mg/L)
Alfadanga (Faridpur)	Median	7.4	0.3	0.6	400	0.033
	IQR	0.3	0.4	2.5	150	0.063
	Mean ± SD	7.4 ± 0.4	0.3 ± 0.2	2.0 ± 2.9	478.6 ± 210.0	0.047 ± 0.034
Kendua (Netrokona)	Median	7.4	0.2	0.5	200	0.007
	IQR	0.4	0.4	1.4	100	0.01
	Mean ± SD	7.6 ± 0.4	0.3 ± 0.3	1.1 ± 1.7	197.0 ± 243.0	0.031 ± 0.053
Shibchar	Median	7.4	0.9	2	450	0.045
	IQR	0.4	0.9	3.9	500	0.028
	Mean ± SD	7.4 ± 0.3	0.9 ± 0.6	2.0 ± 2.8	584.4 ± 352.1	0.057 ± 0.037
Rupsha (Khulna)	Median	7.4	0.2	1.4	1050	0.008
	IQR	0.3	0.1	2.4	1050	0.013
	Mean ± SD	7.4 ± 0.2	0.2 ± 0.1	2.3 ± 2.0	1180 ± 723.0	0.009 ± 0.006
Debhata (Satkhira)	Median	7.4	0.2	0.8	400	0.003
	IQR	0.2	0.2	0.5	150	0.001
	Mean ± SD	7.5 ± 0.4	0.3 ± 0.3	1.4 ± 1.6	391.7 ± 79.3	0.006 ± 0.006
Patharghata (Barguna)	Median	7.4	0.2	0.6	100	0.004
	IQR	0.4	0.2	0.4	400	0.000
	Mean ± SD	7.4 ± 0.3	0.2 ± 0.1	0.9 ± 0.7	717.5 ± 1133.2	0.004
Rangabali (Patuakhali)	Median	7.4	0.1	0.4	500	0.005
	IQR	0.2	0.1	0.2	100	0.002
	Mean ± SD	7.5 ± 0.3	0.1 ± 0.1	0.4 ± 0.3	557.5 ± 84.4	0.004 ± 0.001
Anwara (Chittagong)	Median	-	0.2	2.0	-	0.006
	IQR	-	1.5	3.2	-	0.008
	Mean ± SD	-	0.7 ± 0.8	2.8 ± 3.2	-	0.009 ± 0.008
Bijoynagar (B.Baria)	Median	7.3	0.1	0.3	100	-
	IQR	0.0	0.2	0.9	0	-
	Mean ± SD	-	0.3 ± 0.4	0.6 ± 0.8	-	-
Shajahanpur (Bogra)	Median	7.4	0.3	0.7	100	-
	IQR	0.5	0.4	0.9	0	-
	Mean ± SD	7.4 ± 0.3	0.4 ± 0.4	1.2 ± 1.4	-	-
Kamlganj (Moulvibazar)	Median	-	0.3	2.3	-	0.075
	IQR	-	0.5	6.2	-	0.000
	Mean ± SD	-	0.4 ± 0.3	5.0 ± 6.2	-	-
Sadar (Kurigram)	Median	7.4	-	-	100	-
	IQR	0.5	-	-	100	-
	Mean ± SD	7.5 ± 0.4	-	-	135.0 ± 62.2	-
Bangladesh standard		6.5-8.5	0.1	0.3-1.0	150-600	0.05
WHO standard		6.5-8.5	0.4	0.3	250	0.01

Water pH in shallow tubewell: median (7.4), IQR (0.5), mean (7.5), SD (0.38). Water pH in deep tubewell: median (7.4), IQR (0.4), mean (7.4), SD (0.32)

### Manganese concentrations in drinking water

In our samples, manganese concentrations varied between 0.1 and 5.5 mg/L with a median value of 0.2 mg/L (Table 3). At most sample sites, the median value exceeded the Bangladesh standard of 0.1 mg/L, except Rangabali (Patuakhali) and Bijoynagar (B.Baria). The highest median value (0.9 mg/L) was observed in Shibchar (Madaripur), which exceeded the WHO standard of 0.4 mg/L. Exposure to manganese in drinking water according to the household member characteristics is shown in Table 4. High exposure levels exceeding standards (0.1 mg/L) were found in Chittagong (27 %), Barisal (23 %), and Sylhet (19 %). Those belonging to the lowest wealth group (26 %) had higher exposure to manganese (>0.1 mg/L) than those in the highest wealth group (16 %). When the WHO standard of 0.4 mg/L was considered, the majority of households (82 %) were within acceptable limits. According to Bangladesh standards, about half (51 %) of the households exceeded acceptable limits (>0.1 mg/L).

**Table 4:** Status of chemical parameters by WHO and Bangladesh drinking water standard (%)

Characteristics	WHO drinking water standard (mg/L)								Bangladesh drinking water standard (mg/L)							
	Mn		Fe		NaCl		As		Mn		Fe		NaCl		As	
	≤0.4	>0.4	≤0.3	>0.3	≤250	>250	≤0.01	>0.01	≤0.1	>0.1	≤1.0	>1.0	≤600	>600	≤0.05	>0.05
Sex																
Male	50.3	52.8	50.8	50.8	51.7	50.3	50.0	52.3	50.3	51.2	51.8	48.9	51.9	46.1	50.2	56.7
Female	49.7	47.2	49.2	49.2	48.3	49.7	50.0	47.7	49.7	48.8	48.2	51.1	48.1	53.9	49.8	43.3
*p* value	0.462		0.995		0.643		0.691		0.709		0.290		0.109		0.495	
Age(years)																
≤4	7.1	8.3	8.7	7.0	7.2	7.1	7.1	6.5	6.5	7.8	4.6	6.9	7.2	7.0	7.0	6.7
5-20	35.6	30.9	37.0	34.3	35.0	35.7	35.6	39.3	37.4	32.5	34.5	34.5	35.5	34.9	35.1	53.3
21–40	30.4	32.8	28.7	31.3	32.0	32.1	31.8	23.4	29.2	32.3	30.3	32.2	31.9	32.8	30.1	20.0
41–60	20.0	21.5	18.9	20.5	20.2	17.9	17.2	24.3	20.4	20.2	20.9	18.9	19.0	17.9	19.3	20.0
≥61	7.0	6.4	6.8	6.9	5.6	7.2	8.4	6.5	6.5	7.2	6.7	7.5	6.4	7.4	8.5	90.0
*p* value	0.6623		0.720		0.707		0.358		0.318		0.805		0.975		0.199	
Educational level																
No education	7.1	9.8	8.8	7.3	9.5	6.8	7.5	5.0	5.7	9.4	8.0	7.1	8.0	7.1	6.9	5.0
Primary	20.0	21.2	19.2	20.4	25.9	22.2	15.0	28.3	19.9	20.7	20.9	18.9	26.3	12.7	17.9	30.0
Secondary	20.6	26.5	14.4	22.8	17.2	21.2	21.1	20.0	19.4	23.8	19.5	24.2	19.2	21.4	20.8	20.0
Higher secondary and above	52.2	42.4	57.6	49.4	47.5	49.8	56.4	46.7	55.1	46.2	51.7	49.8	46.5	58.7	54.3	45.0
*p* value	0.166		0.152		0.257		0.175		0.032^**^		0.443		0.010^**^		0.623	
Household economic status																
Ultra-poor	30.1	32.2	24.4	31.8	21.3	19.1	26.4	27.5	27.7	32.8	30.2	30.4	20.3	18.5	25.9	36.7
Poor	36.1	42.7	36.1	37.5	42.3	38.6	33.5	42.2	39.0	35.8	36.0	38.3	41.9	31.9	33.6	63.3
Non-poor	33.8	25.1	39.5	30.7	36.4	42.3	40.1	30.3	33.3	31.4	33.8	31.3	37.7	49.6	40.5	0.0
*p* value	0.018^**^		0.010^**^		0.107		0.168		0.097^*^		0.551		0.003^***^		0.000^***^	
Access to media at home																
No access to media	48.4	50.9	35.7	51.7	82.6	58.3	65.3	35.8	46.6	51.2	52.7	40.8	66.2	76.7	56.1	56.7
Access to media	51.6	49.1	64.3	48.3	17.4	41.7	34.7	64.2	53.4	48.8	47.3	59.2	33.8	23.3	43.9	43.3
*p* value	0.447		0.000^***^		0.000^***^		0.000^***^		0.070^*^		0.000^***^		0.002^***^		0.950	
NGO membership																
No membership	54.0	57.3	56.4	54.2	60.7	59.3	58.7	61.5	54.0	55.4	52.2	59.2	58.6	65.5	60.4	50.0
Member of any NGO	46.0	42.7	43.6	45.8	39.3	40.7	41.3	38.5	46.0	44.6	47.8	40.8	41.4	34.5	39.6	50.0
*p* value	0.324		0.513		0.622		0.622		0.579		0.009^**^		0.051^**^		0.265	
Wealth index																
Lowest	22.1	27.0	28.9	21.7	22.6	21.1	38.4	0.0	20.1	25.9	25.0	20.9	22.0	20.4	28.8	0.0
Second	20.2	21.7	17.3	21.2	18.3	19.1	12.2	9.2	15.6	24.8	21.5	18.2	17.2	25.8	11.1	13.3
Middle	19.0	12.4	19.2	17.5	31.2	24.3	28.3	20.2	21.2	14.7	20.9	13.2	26.6	29.4	26.9	13.3
Fourth	17.0	20.6	16.9	17.8	20.2	17.2	5.5	48.6	17.1	18.2	16.4	19.8	20.0	11.3	15.2	60.0
Highest	21.6	18.4	17.7	21.8	7.7	18.3	15.6	22.0	26.0	16.4	16.2	28.0	14.2	13.1	18.0	13.3
*p* value	0.031^**^		0.066^*^		0.000^***^		0.000^***^		0.000^***^		0.000^***^		0.004^***^		0.000^***^	
Water sources by type																
Shallow tubewell	38.2	78.3	72.9	39.4	74.2	28.4	31.0	70.6	43.0	47.8	42.9	47.2	52.8	21.1	40.2	76.7
Deep tubewell	50.0	21.7	27.1	48.8	1.8	68.6	69.0	29.4	49.4	40.4	42.2	50.9	33.1	78.9	59.8	23.3
Others	11.8	0.0	0.0	11.8	24.1	2.9	0.0	0.0	7.6	11.8	14.9	1.9	14.1	0.0	0.0	0.0
*p* value	0.000^***^		0.000^***^		0.000^***^		0.000^***^		0.000^***^		0.000^***^		0.000^***^		0.000^***^	
Division																
Dhaka	6.6	12.7	11.7	6.8	36.0	40.1	9.1	67.9	6.5	8.9	7.4	8.5	38.1	39.7	22.7	76.7
Chittagong	25.4	39.7	50.8	23.0	1.6	0.0	16.5	9.2	28.7	26.9	27.2	28.4	0.8	0.0	15.6	0.0
Rajshahi	9.3	17.2	10.2	10.8	2.7	0.0	-	-	8.3	13.1	10.8	9.0	1.4	0.0	-	-
Khulna	9.5	1.5	0.0	9.8	1.0	19.8	20.2	16.5	6.5	9.7	7.1	9.5	9.0	25.9	20.9	0.0
Barisal	32.3	5.2	20.3	29.0	24.1	38.5	54.1	0.0	32.9	22.5	41.2	6.9	32.2	34.5	40.8	0.0
Sylhet	16.9	23.6	7.1	20.4	34.6	1.7	0.0	6.4	17.2	19.0	6.3	37.6	18.5	0.0	0.0	23.3
*p* value	0.000^***^		0.000^***^		0.000^***^		0.000^***^		0.000^***^		0.000^***^		0.000^***^		0.000^***^	
HH^a^ member (%)	82	18	17.8	82.2	40.6	59.4	68.9	31.1	48.7	51.3	61.4	38.6	81.6	18.4	91.5	8.5
HH (%)	82	18	18	82	41	59	69	31	49	51	61	39	82	18	91.5	8.5

*p < 0.10; **p < 0.05; ***p < 0.01

^a^Household

### Iron (Fe) in drinking water

The median iron concentration values in water exceeded WHO standards (0.3 mg/L) at all sample sites except Bijoynagar. The median iron values at a few sites exceeded Bangladesh limits (1.0 mg/L) (Table 3). The highest median iron concentration value was in Kamalganj (Moulvibazar) (2.3 mg/L) followed by Anwara (Chittagong) (2.0 mg/L), Shibchar (Madaripur) (2.0 mg/L), and Rupsha (Khulna) (1.4 mg/L). The lowest median value was observed in Bijoynagar (B.Baria) (0.3 mg/L).

About 7 % of young children (≤4 years) were exposed to iron levels in drinking water that exceeded WHO and Bangladesh standards. The highest exposure levels, exceeding the WHO’s acceptable limit of 0.3 mg/L, were in Barisal (29 %) followed by Chittagong (23 %) and Sylhet (20 %) (Table 4). In Dhaka, only 7 % of household members were exposed to greater than 0.3 mg/L iron in drinking water. Only 18 % households met WHO standards (≤0.3 mg/L), while a large proportion (82 %) were exposed to high concentrations of iron in drinking water (>0.3 mg/L). The median iron concentration in deep tubewells was slightly higher (0.8 mg/L)than in shallow tubewells (0.7 mg/L), although median values in both cases exceeded WHO and lower limit of Bangladesh standards.

### Salinity (NaCl) levels

Division-wise variations in sodium chloride levels in drinking water are shown in Table 4. The highest proportion of household members exposed to more than 600 mg/L sodium chloride was found in Dhaka (40 %) followed by Barisal (35 %) and Khulna (26 %). Considering Bangladesh standards (upper limit 600 mg/L), more females than males exceeded their exposure limits (54 % vs. 46 %) (Table 4). As shown in Table 3, excess sodium chloride was detected in Rupsha (Khulna) (1050 mg/L) when the upper limit of Bangladesh standard (600 mg/L) was considered.

### Arsenic (As) concentrations in drinking water

Arsenic testing in the field revealed high arsenic concentrations exceeding Bangladesh standards in Shibchar (Madaripur), Biswanath (Sylhet), and Dhaka. A random sub-sample (over 10 %) was selected for laboratory validation, which showed that water samples collected from Shibchar (Madaripur) (median 0.05 mg/L) and Alfadanga (Faridpur) (median 0.03 mg/L) showed higher arsenic concentrations compared to other sample sites exceeding WHO standard (Table 3). About 68 and 77 % of household members in the Dhaka division were exposed to higher levels of arsenic with respect to WHO (0.01 mg/L) and Bangladesh standards (0.05 mg/L), respectively (Table 4).

### Water Quality Index (WQI)

Drinking water was considered excellent in Kurigram Sadar and Rangabali (Patuakhali) (WQI value < 50) (Table 5). Of the total sample sites, 33 % (4 out of 12 sites) had good-quality drinking water (WQI value < 100) and the majority (67 %) had poor-quality drinking water (WQI value > 100). Quality of drinking water was found very poor in Anwara (Chittagong) and Kamalganj (Moulvivazar), while water was categorized as unsuitable for drinking only in Shibchar (Madaripur).

**Table 5:** Computed water quality values for sample sites

Sample site *upazila* name (district name)	WQI value	Water quality classification based on computed WQI values in sample sites
		<50 = excellent; 50–100 = good water; 101–200 = poor water; 201–300 = very poor water, >300 = water unsuitable for drinking
Rangabali (Patuakhali)	40.05	Excellent water
Sadar (Kurigram)	11.79	Excellent water
Rupsha (Khulna)	92.14	Good water
Patharghata (Barguna)	75.35	Good water
Alfadanga (Faridpur)	169.44	Poor water
Kendua (Netrokona)	142.51	Poor water
Debhata (Satkhira)	113.18	Poor water
Shajahanpur (Bogra)	135.67	Poor water
Bijoynagar (B.baria)	111.83	Poor water
Anwara (Chittagong)	253.29	Very poor water
Kamalganj (Moulvibazar)	258.36	Very poor water
Shibchar (Madaripur)	371.50	Water unsuitable for drinking

## Discussion

Assessment of drinking water quality is a timely requirement amid emerging public health problems in this context where availability of safe water is at risk due to natural and man-made activities. This cross-sectional study conducted across the country aimed at measuring drinking water quality using WQI which delivered messages on the composite effect of chemical parameters on water. The present study is a fact finding or exploratory study contributing to designing and improving program interventions which cover a larger population including high arsenic, high saline prone coastal areas. There is duality about spatial and temporal variations of some chemical parameters. A periodic assessment on arsenic concentration depicts no association with seasonal variations, while repeated assessment of arsenic contents in water based on seasons is assumed to bring little value in health surveillance [18]. In contrast, seasonal and spatial variations of arsenic concentrations in groundwater have been reported by Shrestha et al. [19].

The study findings revealed that drinking water was slightly alkaline, although the ideal pH for human consumption is stated to be 7.4 [20]. A controlled pH of water is suggested in WHO guideline to reduce the corrosion and contamination of drinking water having health consequences. Water pH is influenced by a number of factors including rock and soil composition and the presence of organic materials or other chemicals. Napacho and Manyele [21] found that pH values in shallow tubewells varied between 6.7 and 8.3 due to dissolved minerals from the soil and rocks. They further explained higher alkalinity by the presence of two common minerals, calcium and magnesium, affecting the hardness of the water. On the other hand, water with low pH values is meant to be acidic, soft, and corrosive.

The median value of manganese concentrations exceeded Bangladesh standard at most of the study sites. Other Bengali studies have reported higher manganese levels in drinking water in terms of WHO standards [22]. For example, Islam et al. [23] reported that 52 % of pond-sand filter and 45 % of pond water exceeded Bangladesh drinking water standards. The median value at our sample sites was relatively lower than some previous findings (about 0.8 and 0.9 mg/L) [24, 25] but higher than the 0.1 mg/L reported by Bouchard [26].

Children are reported to be particularly vulnerable to higher manganese concentrations due to their low protective mechanisms. Approximately 8 % of children were exposed to excess manganese concentrations that exceeded both WHO and Bangladesh standards (>0.4 and >0.1 mg/L, respectively). We found higher exposure to manganese in lowest wealth group. This finding has similarity with the other study conducted in Araihazar, Bangladesh [27]. Less exposure among the infants was reported by mothers who had access to TV. Besides, participants living in poor-quality housing type (mud vs. concrete) were more likely to report exposure among the infants. Several studies have reported that exposure to high manganese concentrations threatens children’s cognitive [28], behavioral, and neuropsychological health [25]. However, the potential impact of lower exposure and interactions with other metals are less well characterized. Infants and children are reported to be more susceptible to manganese toxicity than adults [27], and a number of Bangladesh studies have shown that children’s intellectual function, and consequently their academic achievement, was adversely affected by manganese exposure in drinking water [22, 25, 27]. Contradictory to these findings, a higher manganese level in drinking water was shown to be protective against fetal loss during pregnancy of undernourished women in Bangladesh [29].

In most of the sample sites (9 out of 12 sites), iron content in drinking water exceeded upper acceptable limit (1.0 mg/L) of Bangladesh standard. A previous study in rural Bangladesh revealed 50 times higher iron concentrations (mean value 16.7 mg/L) in ground water than WHO’s limit (0.3 mg/L) and reported that 47 % of women consumed above the daily limit of iron (45 mg), likely to increase the risk of health problems [30]. Consumption of >30 mg of iron per day in drinking water was associated with a reduced risk of anemia in individuals without thalassemia [31]. In Gaibandha, half of female respondents consuming >42 mg of iron from drinking water stayed within tolerable limits. If this limit were exceeded, however, the populations would be likely to experience health-related problems including gastrointestinal distress, zinc absorption, and others [32].

Approximately 2 % of women in developed countries but 50 % in developing countries are anemic, contributing to high rates of maternal mortality in developing countries [33]. Iron-deficiency anemia is one of the top ten contributing factors to the global burden of diseases and is considered a public health problem with a high risk of morbidity and mortality in pregnant women and young children [34]. In our study, about half of the female participants were exposed to higher iron concentrations in drinking water which exceeded both WHO and Bangladesh standard. The health impacts of exceeding recommended WHO levels of chemical substances such as iron are often not well documented [32]. There is a duality to iron concentrations: on the one hand, iron deficiency can cause anemia and fatigue, while on the other, excess iron can cause multiple organ dysfunction (e.g., liver fibrosis and diabetes) [35]. In a 10-year period of study in Bangladesh, the prevalence of anemia in women of reproductive age ranged between 23 and 95 % depending on age, pregnancy status, and residency. However, more recent studies have reported iron deficiency as the most important determinant of 7 to 60 % of anemia cases in Bangladesh [36].

Salinity in drinking water was found higher (>600 mg/L) only in Rupsha (Khulna) and Patharghata (Barguna). Geographically, these two *upazilas* are coastal areas. Salinity problems in coastal regions are assumed to be the effects of climate change [37], although industrial and domestic wastes [38] and geological and soil characteristics [21] are also thought to contribute. Bangladesh is at the forefront of the negative effects of climate change and has faced dramatic rises in sea level over the last three decades. Approximately 20 million people living in coastal Bangladesh [24] are dependent on tubewells, rivers, and ponds for drinking water, and these sources are increasingly becoming saline due to rising sea levels [39]. Salinity has intruded over 100 km inland from the Bay of Bengal with consequent health impacts: in a 2008 survey, higher rates of preeclampsia and hypertension were reported in the coastal than non-coastal population [40]. Consistent with this, Khan et al. [41] reported that hypertensive disorders were associated with salinity in drinking water. Furthermore, reducing salt consumption from the global estimated levels of 9–12 g/day [42] to an acceptable limit of 5 g/day [43] would be predicted to reduce blood pressure and stroke/cardiovascular disease by 23 and 17 %, respectively [44].

Most households in Dohar, Shibchar, and Sonargaon used shallow tubewells for drinking, which were affected by high levels of arsenic. In Shibchar (West Kakor village), most tubewells were affected by arsenic, and the villagers were unaware of which tubewell was arsenic free; therefore, they collected drinking water from any tubewell. In some cases (e.g., Sonargaon), people used arsenic-affected drinking water sources even though they knew that the water was contaminated and damaging to health. Bladder cancer risk is increased 2.7 and 4.2 times by arsenic exposure of 10 and 50 μg/L in water, respectively. In this study, there was an 83 % chance of developing bladder cancer and a 74 % probability of mortality at a 50 μg/L exposure level. Mortality rates are 30 % higher at 150 than 10 μg/L [45]. According to a national survey conducted in 2009 by UNICEF/BBS (2011), 53 and 22 million people were exposed to arsenic according to WHO and BDWS standards, respectively. Arsenic has been detected in the groundwater of 322 *upazilas* (sub-districts) and 61 districts in Bangladesh [46]. The health effects of prolonged and excessive inorganic arsenic exposure include arsenicosis, skin diseases, skin cancers, internal cancers (bladder, kidney, and lung), diabetes, raised blood pressure, and reproductive disorders [47].

The overall suitability of drinking water was assessed using a combined measure of water quality parameters: the WQI. The chemical parameters (pH, iron, manganese, salinity, and arsenic) of water samples were used to calculate the WQI value at each site. We applied the weighted arithmetic WQI method to calculate WQI values. In this method, the permissible WQI value for drinking is considered to be 100, the water quality being considered poor if the value exceeded this acceptable limit. Water quality was found excellent only in Rangabali (Patuakhali) and Kurigram Sadar. The water was considered excellent at these sites mainly due to low chemical parameter values contributing to lower composite effect on drinking water quality. In Shibchar (Madaripur), water was categorized as unsuitable for drinking, mainly due to high manganese and arsenic levels found in water at these sites. At most sample sites (e.g., Alfadanga, Kendua, Debhata, Shajahanpur, and Bijoynagar), water was classified as “poor” for drinking due to high manganese values. Moreover, arsenic was also found to be high in Alfadanga (Faridpur) and Kendua (Netrokona). However, in Anwara (Chittagong) and Kamalganj (Moulvibazar), the chemical parameter values in the water samples were very high and contributed to very poor-quality drinking water.

Most respondents at the sample sites used shallow tubewells to obtain drinking water due to lower installation costs. In some areas, such water from shallow tubewells was reported to have high iron and arsenic levels. In coastal districts such as Barguna, Satkhira, and Khulna, water from both shallow and deep tubewells were salty, as reported by the respondents. Yisa and Jimoh [16] reported higher levels of iron and manganese that contributed to poor-quality drinking water. These characteristics are consistent with unplanned waste disposal, agricultural run-off including pesticide or fertilizer, and other environmentally hazardous activities polluting surface water [48].

The study had some limitations. This study embraced cross-sectional study design. However, it would have been better to collect samples throughout the year addressing seasonality and depth of wells. We could not collect data on other WHO-recommended parameters which was beyond our scope of work. Therefore, the analysis has been limited to few water parameters as the requirement of BRAC WASH program and due to resource constraints. Measuring other WHO-recommended chemical parameters might have been a future concern for the program. In addition, water pH would have been tested on the spot using pH meter which was not possible for this study due to limited resources. The limitations observed in this study highlight the insights of future scope of work for research divisions and WASH program.

## Conclusions

Here, we report that drinking water in Bangladesh was mainly alkaline with pH values within acceptable limits. According to WHO standards, a greater proportion of household members are exposed to excessive amounts of iron compared to manganese (82 % vs. 18 %). About half of households exceeded acceptable limits of manganese exposure when considering Bangladeshi standards. Majority of the households used poor quality of drinking water according to WQI values. Higher values of iron, manganese, and arsenic reduced drinking water quality. Awareness raising on chemical contents in drinking water at household level is required to improve public health.

## Abbreviations

As: arsenic
BRAC: Bangladesh Rural Advancement Committee
CDC: Centers for Disease Control
EQ: econo quick
Fe: iron
IQR: interquartile range
MDG: Millennium Development Goals
Mn: manganese
NaCl: sodium chloride
NGO: non-government organization
UNICEF: United Nations International Children’s Emergency Fund
WASH: Water, Sanitation and Hygiene
WHO: World Health Organization
WQI: Water Quality Index
